# Community‐scale models of microbiomes: Articulating metabolic modelling and metagenome sequencing

**DOI:** 10.1111/1751-7915.14396

**Published:** 2024-01-20

**Authors:** Klara Cerk, Pablo Ugalde‐Salas, Chabname Ghassemi Nedjad, Maxime Lecomte, Coralie Muller, David J. Sherman, Falk Hildebrand, Simon Labarthe, Clémence Frioux

**Affiliations:** ^1^ Quadram Institute Bioscience Norwich UK; ^2^ Earlham Institute Norwich UK; ^3^ Inria, University of Bordeaux, INRAE Talence France; ^4^ University of Bordeaux, CNRS, Bordeaux INP, LaBRI, UMR 5800 Talence France; ^5^ INRAE STLO¸University of Rennes Rennes France; ^6^ INRAE, University of Bordeaux, BIOGECO, UMR 1202 Cestas France

## Abstract

Building models is essential for understanding the functions and dynamics of microbial communities. Metabolic models built on genome‐scale metabolic network reconstructions (GENREs) are especially relevant as a means to decipher the complex interactions occurring among species. Model reconstruction increasingly relies on metagenomics, which permits direct characterisation of naturally occurring communities that may contain organisms that cannot be isolated or cultured. In this review, we provide an overview of the field of metabolic modelling and its increasing reliance on and synergy with metagenomics and bioinformatics. We survey the means of assigning functions and reconstructing metabolic networks from (meta‐)genomes, and present the variety and mathematical fundamentals of metabolic models that foster the understanding of microbial dynamics. We emphasise the characterisation of interactions and the scaling of model construction to large communities, two important bottlenecks in the applicability of these models. We give an overview of the current state of the art in metagenome sequencing and bioinformatics analysis, focusing on the reconstruction of genomes in microbial communities. Metagenomics benefits tremendously from third‐generation sequencing, and we discuss the opportunities of long‐read sequencing, strain‐level characterisation and eukaryotic metagenomics. We aim at providing algorithmic and mathematical support, together with tool and application resources, that permit bridging the gap between metagenomics and metabolic modelling.

## Graphical abstract

With this review we articulate two important topics in the context of deciphering the role and functions of microbial communities: metabolic modeling and metagenomics. We survey the methodological approaches, highlight the changes raised by third generation sequencing, and provide resources to bridge the gaps between the sequencing reads and the models.

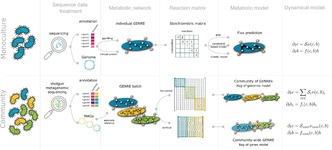

## INTRODUCTION

The roles of microbiomes in the environment and in health‐associated, agricultural or biotechnological applications are widely acknowledged (Foundation et al., 2022; Peixoto et al., 2022; Wilmes et al., 2022). These assemblies of microorganisms are governed by the interactions they have with their environment, and by those occurring among species (Zengler & Zaramela, 2018). Such ecological interactions can generally be classified as neutral, positive or negative, and can be inferred through network analysis (Faust & Raes, 2012). Mechanistically, they operate at a similar level, called the *metabolism*. Metabolism encompasses all of the functions of cells, defined by biochemical reactions and by molecular exchanges between cells and the environment. Microbiomes are characterised by their biosynthetic potential and by the taxonomic diversity that they harbour (Heintz‐Buschart & Wilmes, 2018). While some microbiomes such as the gastrointestinal tract in humans have been extensively studied, thereby revealing insights into the putative cooperation and competition networks among bacteria (Coyte & Rakoff‐Nahoum, 2019), in general, the precise mechanisms, dynamics and control levers of metabolic interactions remain poorly understood. These difficulties are even more prevalent in complex environmental microbiomes where the massive diversity of micro‐organisms and their poor representation in public databases impede taxonomic and functional surveys (Brown et al., 2021).

Culture‐independent methods can be used to broadly characterise microbiomes (Zhang et al., 2019). Prominent among these is metagenomics, the random sequencing of all DNA in a given sample. Metagenomics is by now ubiquitously applied to decipher both the taxonomic and functional diversity of samples; a rich resource of public available metagenomes can support large‐scale studies available to even small labs with the required bioinformatics expertise (Gurbich et al., 2023). Analysis of metagenomic data by bioinformatic tools can generate surveys of the functions embodied by the community (Kindler et al., 2021). Metabolic models, either mechanistic models such as those reconstructed from genome‐scale metabolic network reconstructions (GENREs) or phenomenological models describing phenotypic traits, go further than these surveys. The latter have been the modeller's choice for narrowing down the complexity of community‐wide behaviour (van den Berg et al., 2022), whereas the former could originally only be applied to single species (Varma & Palsson, 1994). However, the range of modelling tools has strongly increased during the past years and now provides a broad collection of methods for answering critical questions about microbial communities (Kumar et al., 2019): What are the roles of bacterial species in the ecosystem? What functions are carried and how redundant are they? How will the community behave over time? And, can we alter this behaviour or modify the community composition to reach a desired state?

Recent developments in metabolic model inference facilitate this procedure and make it worthwhile to consider systematic reconstruction for collections of genomes (Frioux et al., 2020). Concurrently, algorithmic resources for obtaining such genomes from metagenomes have increased, and the technological development of third‐generation sequencing (TGS) has paved the way to a major change of paradigm in the study of microbiomes (Sereika et al., 2022). Our goal here is twofold: (1) to review to what extent metabolic modelling and metagenomics can interact in service of better comprehending microbial ecosystems, (2) to provide comprehensive lists of practical tools that can be used by non‐specialists for metagenomics assembly, metabolic reconstruction and microbiome modelling. We describe the different kinds of models that can be built from metagenomic data to predict the functions and the dynamics of a microbial community, and how they can be used to infer interactions among species. As these models rely on reconstructions of metagenomes, we present as well the latest sequencing techniques, bioinformatic tools and open challenges related to metagenomics, in an attempt to draw together these two domains of computational biology.

## FROM GENE FUNCTIONS TO METABOLIC RECONSTRUCTIONS

Models of microbial communities can be derived from a subset of functions, reactions or metabolic pathways, or on the contrary by analysing the entire metabolic potential of the genomes. In the latter case, the exploration of the link between the genome, enzymes and the metabolic reactions they catalyse provides a system‐wide understanding of the physiology of the cell and its links with its environment. *Genome‐scale metabolic network reconstructions* (GENREs) contain metabolic reactions predicted from the entire genomic content through gene–protein–reaction (GPR) relationships, but also spontaneous reactions and transport reactions connecting the cell to its environment or even to intracellular components (Thiele & Palsson, 2010). In this section, we present the basis of such metabolic networks, whose fundamental concept is the association of genes to functions. Since GENREs are at the heart of mechanistic models of metabolism, the first step of community metabolic modelling is typically the acquisition of a GENRE for each of its members.

GENREs have been built for more than 20 years, with a spectacular acceleration of the publications over the past years, as a result of both the increased availability of genomes and of the efforts of the community for tool development. In 2019, a review tallied more than 6000 published GENREs, 94% of which were for bacteria (Gu et al., 2019). However, the numbers rise even more when including systematic and automatic reconstructions performed on publicly available genomes by platforms such as BioCyc (Caspi et al., 2019) or AGORA2 for human‐associated micro‐organisms (Heinken et al., 2023). Table 1 gathers databases that can be used as general or specialised resources to functionally annotate genomes prior to GENRE generation, as well as collections of metabolic reconstructions. In addition, de novo reconstruction is facilitated by a variety of software programmes and toolboxes created for high‐throughput processing of genomes, an important criterion when tackling large‐scale communities of micro‐organisms. While many tools, catalogued in dedicated reviews (Mendoza et al., 2019), enable this task, only a small number can do so fully automatically, without user interaction. We hereafter present a sample of tools that we believe are suitable for systematic metabolic reconstruction of GENREs starting from a list of annotated genomes.

**TABLE 1:** Main generalist and specialised functional annotation databases and resources of existing metabolic network reconstructions.

Category	Name	Content	Application for metabolic modelling
Generalist databases	KEGG	Several databases including biochemical reactions (Kanehisa et al., 2021)	General annotation of genes and genomes that can be directly or indirectly (via reconstruction tools) associated with metabolic reactions
	COG	Clusters of orthologous genes (Galperin et al., 2020)	
	Gene Ontology	Knowledge base of gene functions (Galperin et al., 2020)	
	UniProt	Association of proteins to functions (UniProt Consortium et al., 2021)	
	Pfam	Classification of proteins into families and domains (Mistry et al., 2020)	
	BRENDA	Collection of enzymes and their metabolic reactions (Chang et al., 2020)	
	eggNOG	Orthologous groups and functional annotation (Huerta‐Cepas et al., 2018)	
	MetaCyc	Encyclopaedia of metabolic reactions (Caspi et al., 2019)	
Metabolic reconstruction collections	BioCyc	More than 20,000 pathway–genome databases (Karp, Billington, et al., 2019)	Ready‐to‐use reconstructions and models
	BiGG	High‐quality metabolic network reconstructions (King et al., 2016)	
	BioModels	Literature‐associated (metabolic) models (Malik‐Sheriff et al., 2019)	
	AGORA2	GENREs of 7302 human‐associates micro‐organisms (Heinken et al., 2023)	
	EMBL GEMS	GENREs reconstructed with Carveme for 5587 reference and representative bacterial genomes of NCBI RefSeq (Machado et al., 2018)	
Specialised databases	CAZy	Carbohydrate‐active enzymes (Drula et al., 2022)	Curation, validation, inspection of model predictions
	CARD	Antibiotic resistance (Alcock et al., 2020)	
	TCDB	Transporter classification (Saier et al., 2021)	
	Norine	Non‐ribosomal peptides (Flissi et al., 2020)	
	antiSMASH	Natural products of the secondary metabolism (Blin et al., 2020)	
	MEROPS	Proteolytic enzymes and their substrates (Rawlings et al., 2017)	

Gapseq (Zimmermann et al., 2021) builds GENREs using a curated reaction database and a dedicated gap‐filling algorithm. The tool was notably applied to a four‐strain microbial community whose associated models reproduced expected behaviours of the gut microbiome metabolism. Carveme (Machado et al., 2018) uses a top‐down approach consisting in pruning (carving) a universal curated model of the microbial metabolism for rapid generation of GENREs starting from genomes. While construction with Gapseq takes up to 2 h, a Carveme‐generated reconstruction can be obtained in a few minutes. Both computation times are compatible with a systematic generation of GENREs for microbiomes. Pathway Tools (Karp, Midford, et al., 2019) builds pathway–genome databases relying on the MetaCyc database of genomes (Caspi et al., 2019) and on functional annotations, for example, from EggNOG‐mapper (Cantalapiedra et al., 2021), Prokka (Seemann, 2014) or RAST (Overbeek et al., 2014). While Pathway Tools is originally based on an interactive single‐organism interface, which can impede its use at the scale of large communities, it is possible to automatise the process using, for instance, the Python package mpwt (Belcour et al., 2020). The latter was applied to a collection of 336 reference genomes obtained after metagenomic read mapping, in order to identify the presence of pathways of interest (Ruuskanen et al., 2021). The latest developments of Pathway Tools additionally permit importing a set of genomes or metagenome‐assembled genomes (MAGs) (see Section “From Metagenomic Data to Assembled Genomes” below) into the interface to run the reconstruction on each organism. AutoKEGGRec (Karlsen et al., 2018) is a Matlab programme that creates draft GENREs by retrieving reactions and GPR relationships from the KEGG database for one or a set of organisms, making it suitable for community modelling. In addition, online platforms provide reconstruction services, such as KBase (Arkin et al., 2018), which proposes a large variety of analyses including metabolic reconstruction, with extensive efforts towards reproducibility through the use of narratives; or ModelSEED (Seaver et al., 2020), which performs metabolic annotation and GENRE reconstruction for microbes, plants and fungi.

A general limitation of GENREs lies in the metabolic landscape covered in reaction databases: many proteins have no attributed functions even in well‐studied organisms, and experimental evidence of functions is even scarcer (Monk et al., 2014). It must be noted that automatically reconstructed GENREs cannot compete with manually curated ones with respect to the quality of the contents and the accuracy of the simulations they permit. High‐quality GENREs exist mostly for model organisms; automatically reconstructed GENREs may suffer from erroneous GPR relationships or reversibility constraints, and inaccuracy in biomass composition (Gu et al., 2019). Most automated tools produce GENREs that are gap‐filled in order for the associated model to simulate the production of biomass (Feist & Palsson, 2010), which is a common step for flux prediction (see Section “Genome‐Scale Metabolic Modelling” below). Unfortunately, automated gap‐filling, while useful, is not as reliable as manual curation for producing high‐quality GENREs (Karp et al., 2018; Latendresse & Karp, 2018). Additionally, growth is simulated in media that likely do not sustain the auxotrophies of non‐cultivable organisms, which may depend on complex metabolic interactions with other microbes to grow (West et al., 2007; Zengler & Zaramela, 2018). It is possible to work with non‐gap‐filled GENREs, for example, using Pathway Tools or AutoKEGGRec. Overall, the use of simulation‐ready GENREs creates a risk of missing interactions occurring in communities (false‐negative interaction), while using non‐gap‐filled GENREs may falsely predict interactions to sustain certain functions that could actually be performed by individual microbes (false‐positive interaction). We refer to Bernstein et al. (2021) for a description of all the steps of the GENRE reconstruction and simulation processes where uncertainty may occur and lead to inconsistent results. More generally, with the increasing use of metagenomic‐assembled genomes (see Section “From Metagenomic Data to Assembled Genomes” below), more attention is paid to the effect of genome incompleteness on the inference of functions and metabolic potentials (Belcour et al., 2020; Eisenhofer et al., 2023). The examination of multiple GENREs when building models of microbiomes must therefore consider these potential shortcomings and address them through a careful choice of model paradigm and a cautious interpretation of results.

Obtaining a GENRE for each member of a microbial community is already per se a valuable resource for querying and comparing the metabolic processes that can occur within species, even before modelling and simulation. It can consist in identifying functional differences between GENREs: strain‐specific patterns, gene essentiality or biosynthetic capacity (Biggs et al., 2015). In Weiss et al. (2022), GENREs corresponding to the 12 bacterial members of a synthetic bacterial community were compared at the level of metabolic pathway, as a complement to results of in vitro interaction experiments. More generally, any approach aiming at comparing metabolic networks could be applied to GENREs of co‐occurring species (Ay et al., 2012). Likewise, studying the pan‐reactome and accessory reactome of species using GENREs is valuable (Belcour et al., 2023; Prigent et al., 2018) and these approaches can be applied to ecosystems.

## METABOLIC MODELS FOR MICROBIAL COMMUNITIES: FROM GENRES TO DYNAMIC SIMULATIONS

GENREs can be the input of metabolic modelling at the genome or community scale. In this section, we describe and focus on mechanistic constraint‐based models. Alternative GENRE‐based models and their relationship to other modelling fields are presented in Section “Towards Genomics‐Informed Phenomenological Models of Microbial Communities”.

**FIGURE 1:**
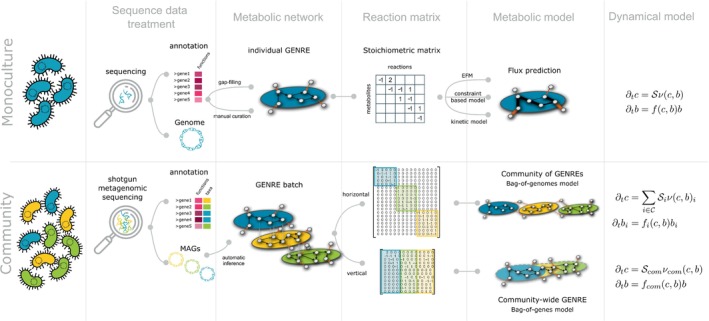
From mono‐organism to community function‐based model of microbial populations. *Monoculture model* (upper panel): in monoculture, a GENRE is obtained from the annotated genome, with semi‐automatic tools (e.g. gap‐filling) and manual curation. A matrix representation of the metabolic network, the *reaction matrix*, is an input for flux prediction models. Metabolic fluxes can be computed iteratively to estimate changes over time of a dynamical model of the population. *Community model* (lower panel): in a microbial community, the metagenome makes it possible to obtain MAGs and GENREs for members of the community. GENREs may share some common functions and metabolites (vertical grey vertices in the GENRE batch). A first paradigm for building a community‐wide metabolic model is a *bag‐of‐genome* model: A set of independent GENREs interacting through common external metabolites. The corresponding reaction matrix is the concatenation of the individual matrices, and the corresponding dynamical model is the sum of the fluxes computed for each unique genome. A second paradigm termed *bag‐of‐gene* model consists in merging all the GENREs through their common functions and metabolites, resulting in a global metabolic network for the whole community. The corresponding reaction matrix is the concatenation of the individual matrices after merging or removal of common reactions (columns) and metabolites (rows), resulting in a lower dimension matrix and a dynamical system with a unique equation. With this paradigm, analysis can be performed at the scale of a community, but the contribution of each taxon is fuzzier.

When modelling a community, the first step is defining a collection of individual GENREs representative of the community. The selection can be ‘reductionist’, meaning a reduced number of genomes is selected as functionally representative of the community and a GENRE is obtained for each, either from de novo reconstruction or from databases (Table 1). A second ‘exhaustive’ approach consists in building a GENRE for each genome encountered in the community, for example, from metagenomic analysis. Then, two alternative conceptual views exist to build a community model, with methodological and computational pros and cons (Figure 1): one consisting in merging GENREs to build *a community‐wide model*, also termed *bag‐of‐genes model* and the other articulating individual GENREs into a *community of GENREs* (*bag‐of‐genomes*) (Frioux et al., 2020).

### Genome‐scale metabolic modelling

#### Metabolic networks, metabolic models and dynamic simulations

A GENRE has a matrix representation termed the *stoichiometric matrix* S, with as many rows as metabolites and as many columns as reactions (Figure 1). The coefficient $S_{\mathrm{ij}}$ is the stoichiometric coefficient of the metabolite *i* in the reaction *j*, with a negative or positive value indicating whether *i* is a substrate or a product of *j*, or a zero value if the metabolite *i* does not appear in reaction *j* (Orth et al., 2010). The matrix S allows modelling the dynamics of the metabolite concentrations *c* (mmol L^−1^) through the mass‐balance equation

(1) $$\partial_{t}c=S\nu \left(c,b\right)$$

where $\partial_{t}$ is the time derivative operator and $\nu \left(c,b\right)$ (mmol h^−1^ L) is a vector of the reaction fluxes that depend both on *c* and on the bacterial population *b (g L^‐1^*) (Jamshidi & Palsson, 2010). Equation (1) highlights that modelling concentration variation comprises two distinct components: (1) the metabolic network, that is, the static functional potential carried by the micro‐organism genome represented by S, and (2) the distribution of the metabolic fluxes on this network, that is, the activated functions in the current state of the system, represented by ν. The resulting model is referred to as a *genome‐scale metabolic model* (GEM). The algebraic analysis of S already gives biological insights into the metabolic graph, through the use of approaches such as elementary flux modes (EFM) (Stelling et al., 2002).

#### Constraint‐based models for flux prediction

To predict ν for a single species or a merged community (see Figure 1), several approaches are available. *Kinetic modelling* adds the enzymes catalysing the reactions to the system and evaluates ν using Michaelis–Menten‐like equations that link substrate and enzyme concentrations to the reaction kinetics (Costa et al., 2016). However, due to the difficulty of model parametrisation, which requires enzyme kinetic rates, practical use is limited at a genome scale (van Rosmalen et al., 2021) and even more so at metagenome scale. *Constraint‐based metabolic models* are a widely used alternative for predicting flux distributions that assumes a quasi steady state of the intracellular metabolites in Equation (1). *Flux Balance Analysis* (FBA) (Orth et al., 2010) solves a linear optimisation of an objective function—usually the biomass production—and taking into account thermodynamic constraints modelled as flux bounds.

Known limitations of FBA are the need for high‐quality or curated GENREs as input, and its sensitivity to the definition of the biomass function, which is organism‐dependent (Xavier et al., 2017) and is hard to establish for a community‐wide model. Another limitation is the existence of many alternative optimal solutions to the problem. Numerous variations exist, such as parsimonious FBA (pFBA) (Lewis et al., 2010), which focuses on sparse flux distributions; flux variability analysis (FVA) (Mahadevan & Schilling, 2003), which explores the range of alternative solutions to identify essential and blocked reactions; thermodynamic flux analysis (TFA) (Henry et al., 2007), which identifies thermodynamically feasible flux distribution; dynamic FBA (dFBA) (Mahadevan et al., 2002), which iterates flux evaluations and concentration updates to model the dynamics of the system; and resource balance analysis (RBA) (Goelzer et al., 2015), which extends the optimisation problem to take into account the internal machinery of protein production. We refer to León and Nogales (2022) for an extensive review of constraint‐based methods discussing the biological trade‐offs they achieve. The Cobra toolbox (Heirendt et al., 2019) and its Python interface (Ebrahim et al., 2013) provide numerous practical tools and implementations for constraint‐based modelling.

Several methods integrate *–omics* data into constraint‐based models, such as transcriptomic (Jenior et al., 2020) or metabolomic data (Toya & Shimizu, 2013). Metatranscriptomics data can support the creation of condition‐specific community metabolic models such as in the work of Zampieri et al. (2023). Going beyond the analysis of functional potential—which may not be expressed—this approach takes into account whole transcriptome abundance of MAGs and gene transcript abundance, to scale and adapt metabolic reaction bounds. Such integrated solutions bring metabolic simulations closer to the biological reality.

Building constraint‐based models using GENREs of individual microbiome members is a first step in the comparison of their biosynthetic potential (Bartell et al., 2014). As one approach, CONGA compares GENREs pairwise to identify essential genes (Hamilton & Reed, 2012). Pangenome analysis combined with flux analysis can also be used to detect strain‐specific metabolic features (Bosi et al., 2016) (see also Section “Metagenomic Strains”). A further step in the analysis is to combine individual GEMs in order to model the community and its interactions.

### From genome to metagenome‐scale metabolic modelling

#### Bag‐of‐genomes models

A batch of GENREs can first be considered as a set of independent individuals interacting through extracellular metabolites. The community model is then obtained by the juxtaposition of the individual GENREs, which are used to predict individual metabolic fluxes, the sum of which determines the fate of the extracellular metabolites (Figure 1). This paradigm is also termed a *bag‐of‐genomes* model (Frioux et al., 2020).

However, the prediction of individual fluxes can require new methodological developments, in particular in a multispecies FBA framework where interactions are studied in a static regime (Gottstein et al., 2016). Indeed, the growth rate of an individual only depends on the availability of extracellular metabolites, whereas that of a community constraint‐based model also depends on the instantaneous use of metabolites by organisms that limits metabolic availability for others. Individual flux prediction in communities is thus the result of a trade‐off of metabolite uptake between the community members. Furthermore, the hypothesis of individual optimal growth, which is sound in monoculture, may not be valid in communities where complex ecological processes may occur. For example, symbiosis can induce suboptimal growth for a symbiont but improve long‐term persistence in the community. To predict this trade‐off, several methods have been developed, that are conceptually very different in a static or a dynamic FBA framework. These methods and example of corresponding toolboxes are recapitulated in Table 2. Extensive reviews of community‐wide metabolic models can be found in Colarusso et al. (2021), including comparison between FBA and EFM‐based methods, in León and Nogales (2022), for a comparison between top‐down and bottom‐up modelling approaches, or in García‐Jiménez et al. (2021) and Zaramela et al. (2021), for a parallel between computational methods and engineered microbial communities.

**TABLE 2:** Main methods and corresponding framework to solve community FBA and dFBA models.

Framework	Method	Description	Platform and references
FBA	Multi‐level optimisation	An inner optimisation is made for each individual GENRE and coupled to an outer community‐wide optimisation: trade‐off between individual and total biomass growth	Optcom (Zomorrodi & Maranas, 2012) CASINO (Shoaie et al., 2015)
	Weighted squared sum	A weighted square sum of individual flux weighted by microbe abundance as community‐scale objective function	MICOM (Diener et al., 2020)
	Multi‐objective FBA	A Pareto front is computed as set of solutions of a multi‐objective optimisation problem. A solution on the Pareto front is chosen according to additional criteria (e.g. resource use)	MO‐FBA (Budinich et al., 2017; Heinken & Thiele, 2015; Heinken et al., 2013)
	EFM‐based	EFM‐based individual models the kinetic rates of which is determined by a community‐scale model	CODY (Geng et al., 2021)
dFBA	Sum of fluxes	Straightforward sum of individual fluxes (see Figure 1). Flux bounds are computed from extracellular compound concentrations that change over time according to consumption and production by community members	*μ*BialSim (Popp & Centler, 2020) COMETS (Dukovski et al., 2021) IndiMeSH (Borer et al., 2019)

The Microbiome Modelling Toolbox (Heinken & Thiele, 2022) derives community models from metagenomics data for the human gut microbiome. This platform facilitates the extraction of GEMs corresponding to OTUs identified by metabarcoding from large curated collections such as AGORA 2 (Heinken et al., 2023) or the virtual metabolic human (Noronha et al., 2018), and provides efficient computation and result visualisation. The main drawbacks of such large bag‐of‐genomes models are the computational cost, which scales with the number of GEMs taken into account (Heinken & Thiele, 2022), and the difficulty of ensuring GENRE quality and accuracy for a large number of genomes. In particular, the presence of draft models inferred by automatic pipelines may impair the overall quality of the simulations (Babaei et al., 2018). Bag‐of‐genomes models can be embedded in an outer optimisation problem in order to find an optimal configuration of the microbial community according to a dedicated fitness function, like in FLYCOP (García‐Jiménez et al., 2018).

#### Bag‐of‐genes models

A community‐wide metabolic model can be built by assembling all the functions carried by the individual GENREs and factoring the common functions shared by different organisms (Figure 1). In this paradigm, the community is seen as a meta or supra organism, an *enzyme soup*, whose functional potential results from the gathering of the whole set of functions (Frioux et al., 2020). The corresponding stoichiometric matrix $S_{\mathrm{com}}$ is the concatenation of the individual stoichiometric matrices after removal of duplicated metabolites and functions.

Bag‐of‐genes models have been compared to the bag‐of‐genomes approach in the context of natural microbial communities in hot spring mats (Taffs et al., 2009). A community‐wide metabolic model has been reconstructed with KBase and compared with expression data in a bacterial photoautotroph–heterotroph consortium (Henry et al., 2016). The same method was reused to reconstruct a bag‐of‐genes model of child gut microbiota for a modelling study of malnutrition‐associated dysbiosis (Kumar et al., 2018).

Carveme (Machado et al., 2018) provides an alternative between a community‐wide GEM and a bag‐of‐genomes model by assigning a compartment to each species and adding an extracellular compartment to connect them. A community biomass equation is inferred, enabling the use of the constraint‐based models described above while keeping the knowledge of metabolite producers. HUMAnN2 can also help navigate between bag‐of‐genomes and bag‐of‐genes models by providing both community and species‐resolved functional potentials from metagenomic data (Franzosa et al., 2018).

## MODELS FOR LARGE‐SCALE METABOLIC EXPLORATION OF MICROBIAL COMMUNITIES

The recurring goals, when considering a microbial community, are first to identify putative interactions among its members, then to globally assess the role of all species in the community. We focus here on models based on GENREs dedicated to the direct inference of putative interactions and describe how discrete models of metabolism can be an alternative to constraint‐based GEMs for scalable screening of metabolic potential in microbial communities.

### Assessing competition and cooperation potentials of microbial communities

**FIGURE 2:**
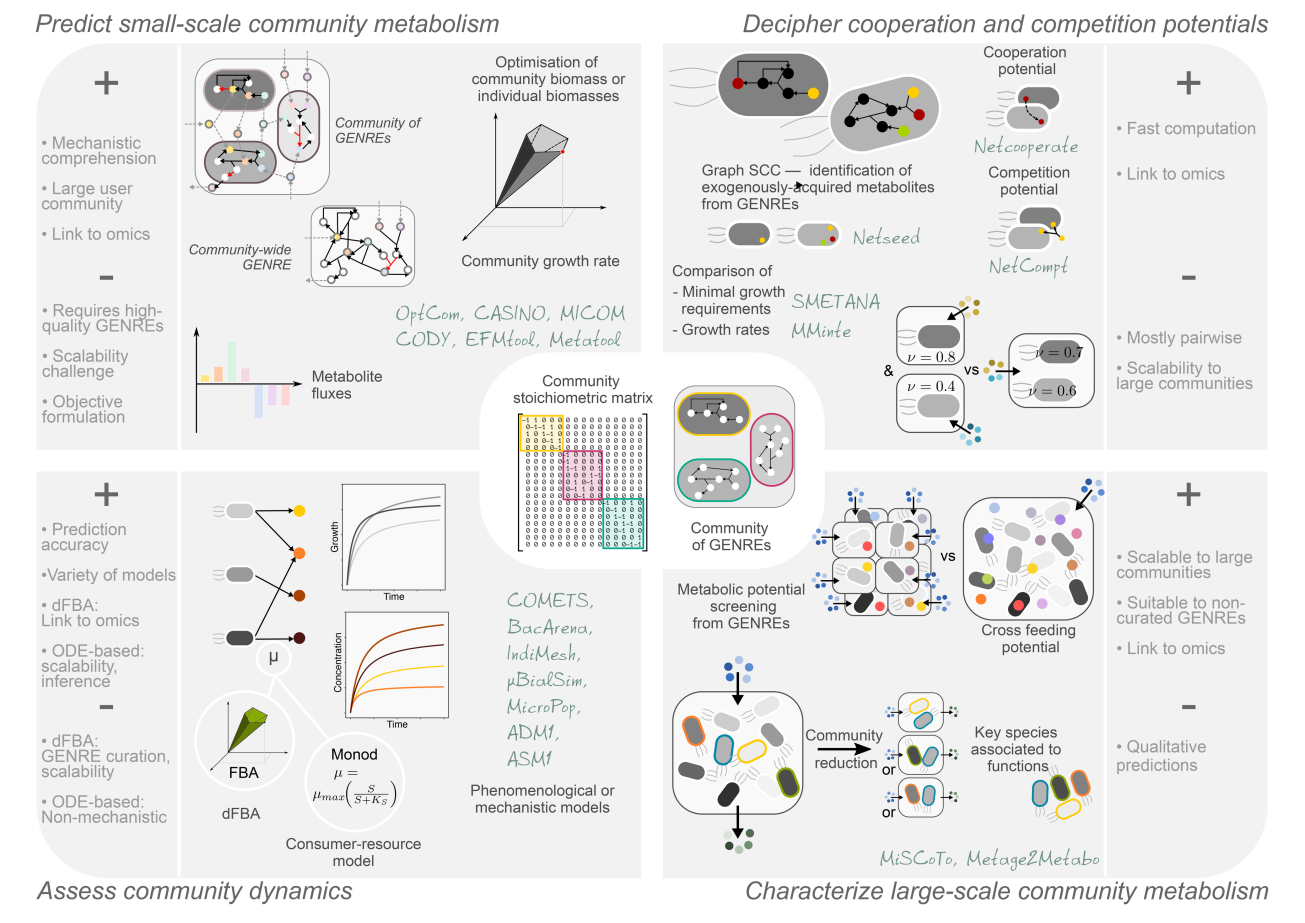
Approaches for numerical simulation and reasoning on community metabolism. Methods and tools are sorted according to the functional characterisation of the microbial community metabolism they can provide: predicting small‐scale community metabolism (upper left panel), assessing community dynamics (lower left panel), deciphering cooperation and competition potentials (upper right panel) and characterising large‐scale community metabolism (lower right panel). For each class, the main methods and dedicated software (grey italic names) are recapitulated. The main ‘pros’ (+) and ‘cons’ (–) are listed on the lateral box associated with each panel.

Cooperation through metabolic exchanges and competition for shared nutrients are the main metabolic interactions that occur in microbial communities. As a result, being able to estimate cooperation and competition potentials is essential for characterising, comparing and selecting communities. Approaches dedicated to this purpose can be separated in two categories, the first one composed of methods that structurally analyse and compare individual GENREs and the second comparing the individual growth rates of bacterial species to those obtained in communities using GEMs (Figure 2 upper right panel).

*Reverse ecology* (Levy & Borenstein, 2012) aims at inferring ecological mechanisms through the characterisation of the composition of the environment and the interactions occurring in it. A core step is the detection of exogenously acquired compounds, or *seeds*, that NetSeed (Borenstein et al., 2008) computes using the GENRE's strongly connected components. NetCooperate (Levy et al., 2015) quantifies the potential for pairwise syntrophic relationships. The *biosynthetic support score* quantifies the proportion of an endosymbiont's seeds occurring in a host's GENRE. The *metabolic complementarity index* of two bacterial species is the fraction of one GENRE's seeds that are products of the second GENRE, and are not in its own seed set. Comparing GENREs pairwise can also be used to determine a competition potential. In Levy and Borenstein (2013), the *metabolic competition index* is the fraction of an organism's seeds that also belong to the seeds of another. NetCompt (Kreimer et al., 2012) calculates the *effective metabolic overlap*, combining seed calculation with the network expansion algorithm (see Section “A Boolean Abstraction of Metabolic Producibility to Scale Up to Large Communities”), and estimating the reduction of a species' metabolic potential in the absence of source compounds that are shared with other species. These metrics have been used for determining a large‐scale evolutionary trend in the loss of biosynthetic capacity over time in parasites (Borenstein & Feldman, 2009), and detecting enzymes operating on exogenously acquired compounds in diseased individuals (Greenblum et al., 2012). They were also integrated in analysis frameworks such as RevEcoR (Cao et al., 2016) or PhyloMint (Lam et al., 2020). It is worth noticing that the seed set is not only the set of nutrients required for growth but includes compounds that may theoretically enable the production of any metabolite in the network, and can therefore underestimate or overestimate interaction potentials (Lam et al., 2020).

Other methods use the computation of flux distributions with constraint‐based modelling to determine scores of competition and cooperation. Freilich et al. (2011) calculate the *potential competition score* and the *potential cooperation score* to characterise metabolic interactions between nearly 7000 combinations of 118 bacterial GEMs. The authors additionally infer win–lose and give‐and‐take relationships between bacterial pairs by assessing their growth and co‐growth rates in competition‐ and cooperation‐inducing media. MMinte (Mendes‐Soares et al., 2016) compares the growth rates of species in pairwise interacting communities to their individual growth rates in order to predict whether one species can benefit from or be negatively impacted by the presence of another. SMETANA overcomes the limitation of pairwise simulation and computes interaction scores for microbial communities by solving mixed integer linear programming problems (Zelezniak et al., 2015). It computes the *metabolic interaction potential,* which represents the extent to which metabolites produced by the community can be reused by species for their growth. The nutrients required for growth in a non‐interacting community can be compared between species, leading to the *metabolic resource overlap*, an estimation of competition potential. Machado et al. (2021) used these scores and described a polarisation of microbial communities between competitive and cooperative metabolisms. Finally, the use of taxon knock‐outs in MICOM (Diener et al., 2020) can also be a means to evaluate the positive or negative impacts of species on the growth of other community members.

### A Boolean abstraction of metabolic producibility to scale up to large communities

As depicted in the above sections, most approaches for modelling interactions in microbial communities are applied pairwise, and more generally, constraint‐based models are not applied to large communities due to both the curation needs for the individual GENREs and the computational costs associated with simulations. This motivates the use of alternative paradigms for modelling. One approach is *metabolic network percolation* (Bernstein et al., 2019), a probabilistic concept that randomly samples environmental compositions to assess the consistency of metabolite production in microbiomes. Applied to the human oral microbiome, it permitted the creation of an atlas describing metabolite biosynthesis potentials while being more tolerant to missing reactions than the classical constraint‐based simulations.

Discrete and reasoning‐based models are further approaches for estimating the ability of a species to synthesise, or not, metabolic compounds from available substrates in its environments. The *network expansion algorithm* (Ebenhöh et al., 2004) provides a Boolean approximation that converts the stoichiometric matrix into a matrix of incidence of 0, 1 and −1, representing solely the participation of molecules in metabolic reactions, hence discarding mass conservation. The metabolic potential of a species, called *scope*, is calculated from a set of available nutrients, the *seeds*: metabolic reaction activation depends on the availability of its reactants either from the seeds or as the products of other activated reactions. MeneTools implements network expansion to query metabolic capabilities of GENREs (Aite et al., 2018).

Metabolic scope can be used to determine the metabolic potential of several organisms in a community (Christian et al., 2007). Ofaim et al. (2017) built a framework considering the relationship between taxonomy, function and environment, and attempted to use metabolic modelling to explain the co‐occurrence of species in the plant rhizosphere or the soil. A similar approach applies to deciphering symbiont–host interactions: Opatovsky et al. (2018) simulated the metabolism of obligatory and facultative symbionts associated with a phloem‐feeding whitefly. As a microbiota can involve hundreds of species living in a shared environment, the scalability of network expansion is a valuable property accounting for characterising the associated metabolic diversity (Figure 2 bottom right panel). The community‐wide implementation of network expansion in MiSCoTo (Frioux et al., 2018) permits computing metabolic potentials of communities and selecting minimal size communities exhibiting functions of interest. Metage2Metabo integrates such analyses together with GENRE reconstruction to systematically compare the metabolic potential of metagenomic samples or genome collections (Belcour et al., 2020). Discrete models of this kind were used to screen microbial metabolic potentials (Ramalho et al., 2022), to predict potential symbiotic relationships between MAGs from patients with Crohn's disease and healthy individuals (Sabater et al., 2022) and to analyse metabolic complementarity (Karimi et al., 2021).

## TOWARDS GENOMICS‐INFORMED PHENOMENOLOGICAL MODELS OF MICROBIAL COMMUNITIES

The use of mechanistic GEMs in the context of large microbial ecosystems can be impeded by poorly reconstructed GENREs (Babaei et al., 2018) or by issues related to the high dimension of the system, such as uncertainties, accumulations, analysis of massive outputs or computational load. A large number of phenomenological microbial community models have proved to be efficient in capturing macroscopic features without involving genome‐scale inputs. In this section, we discuss opportunities for coupling GEMs with phenomenological and hybrid models to achieve scalability for complex microbiome, while allowing genome‐resolved –omics integration.

### Existing models that could benefit from metabolic modelling

Microbial community models other than GEMs have recently been divided into four types: (1) consumer–resource, (2) generalised Lotka–Volterra (gLV), (3) trait‐based models and (4) individual‐based model (IBM) (van den Berg et al., 2022). Models (1)–(3) are population dynamic models based on a system of ordinary differential equations (ODE), describing the rate of change of the population density depending on other species ((2) directly, (1) indirectly), available resources (1) and environmental factors (3). Model (4) describes the fate of individual cells, whose juxtaposition models the population. IBMs have been used on a wide range of ecosystems in order to gain knowledge of the underlying ecology and for decision‐making (DeAngelis & Grimm, 2014). Models of type (4) are not opposed to those of type (1)–(3), as arguably they can be complementary to one another (Hellweger et al., 2016), and since ODE models can be derived as large population approximations of IBM models (Darrigade et al., 2022). Due to their computational efficiency, models (1)–(4) can be spatialised, using, for example, partial differential equations (PDEs) as Labarthe et al. (2019), Moorthy et al. (2015) and Smith et al. (2021) did for the gut microbiota.

GEMs can be considered not only as a category by themselves but can also be included in models of types (1)–(4).

#### Coupling GEMs with consumer–resource models

It must be stressed that bag‐of‐genomes models (see Figure 1) can be interpreted as particular cases of consumer–resource models (Figures 2 bottom left panel and 3), where resource consumption, metabolite production and growth rates are modelled through FBA fluxes. Hence, existing consumer–resource models can be easily supplemented with a genome‐scale metabolic model. As such, a consumer–resource model that qualitatively reproduces experimentally observed ecological patterns of the Earth Microbiome Project by considering the exchange of secondary metabolites (Marsland et al., 2020) could be refined with GEMs: export fluxes for the metabolites included in the consumer–resource model could be computed dynamically with the GEM while remaining essential metabolites would be given a steady‐state value.

**FIGURE 3:**
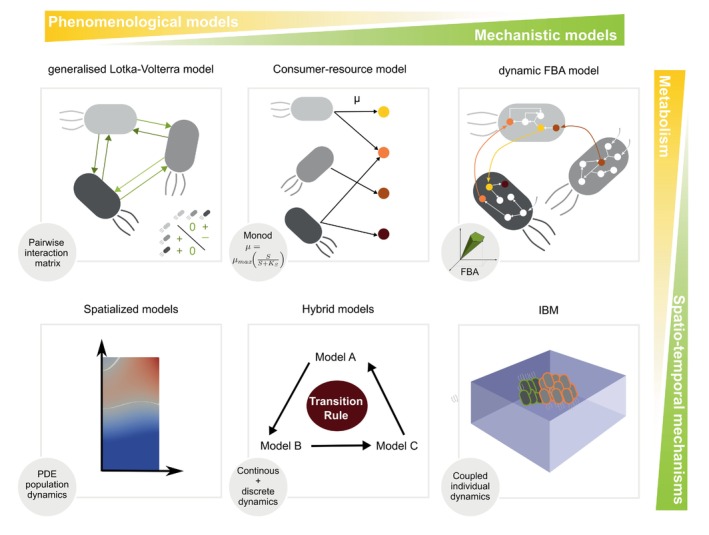
Dynamic models of metabolism: opportunities beyond GENRE‐based models. Different formalisms can be used to capture microbial population dynamics (upper panel). These models can be placed on a gradient, from phenomenological (left) to mechanistic models (right) of the metabolic capabilities of the community. Each formalism can be supplemented by additional spatio‐temporal mechanisms (lower panel) that can be represented at the population (PDE) or individual scale (IBM). The addition of spatio‐temporal features in the community dynamics induces simplifications in the metabolic models to keep the model computationally tractable.

#### Deciphering gLV interactions with GEMs

gLV models proved to be efficient in analysing interactions across the gut microbiome by fitting time series of population counts (Stein et al., 2013). Population data obtained from high‐throughput sequencing reports relative abundances, inducing inference difficulties (Remien et al., 2021). That drawback can be overcome by providing *priors* to the inference methods (van den Berg et al., 2022), for instance from GEMs. In an example coming from anaerobic digestion (AD), GEMs were reconstructed for each of the 800 species present in the community, and FBA was used to understand the syntrophic relations between micro‐organisms (Basile et al., 2020) that could be used as *priors* of gLV model parameters. Conversely, a pairwise interaction identified in time series with a gLV model could be interpreted using pairwise GEMs.

#### Supplementing trait‐based models with GEMs

Bioreactor modelling is a good example that relates to both trait‐based models (since they often involve abiotic parameters such as pH or temperature) and consumer–resource models. Two examples of widely implemented bioreactors are wastewater treatment plants based on activated sludge (AS) (Tchobanoglus et al., 2003), and biogas production from AD of organic waste (Moletta, 2015). AS (Leslie Grady Jr. et al., 1986) and AD (Batstone et al., 2002) systems have been modelled in the framework of community‐wide ODE systems (see Figure 1), representing microbial species as functional guilds, that is, as a population of microbes responsible for a metabolic pathway. AD models have been used in various contexts such as ruminal (Muñoz‐Tamayo et al., 2016), environmental (Desmond‐Le Quéméner et al., 2021) or gut microbiota (Kettle et al., 2018; Muñoz‐Tamayo et al., 2010). Since considerable efforts have been made to sequence microbes present in AD and AS at a global scale (Dueholm et al., 2022), one could readily envision enhancing parts of the AD and AS with metabolic models. However, FBA analysis alone may not suffice to represent abiotic inhibition arising in AD systems (Basile, Zampieri, et al., 2023).

#### Coupling IBM with GEMs

In an IBM model, each individual can be provided with a GEM, which can be used to predict its metabolism with FBA. Such a framework has been already included in platforms like BacArena (Bauer et al., 2017), ACBM (Karimian & Motamedian, 2020), IndiMeSH (Borer et al., 2019) or CROMICS (Angeles‐Martinez & Hatzimanikatis, 2021), that were recently compared (Scott et al., 2023). These frameworks allow spatialisation: the IBM can include movement functions modelling microbial motility, and complex multi‐physics features, for example, the complex diffusive pattern in the rhizospheric area for IndiMesh.

Since coupling system dynamics, IBM and GEMs involve different mathematical formalisms, hybrid models provide a unifying framework that can help implementing model coupling, as described in the next section.

### Extending the scope of community modelling with hybrid models

The complex behaviours of microbiomes arise from the interaction of different functional processes with different dynamics, that can be considered as continuous, discrete or random, depending on their timescale, dictating appropriate mathematical formalisms (Assar et al., 2014): *numerical ODEs* for continuous dynamics, *automata* for discrete instantaneous changes, *non‐deterministic* or *stochastic models* for uncertain or random transitions. This highlights the need for a flexible framework to explore conjointly these processes at the community scale.

*Hybrid models* are such a framework, by assembling modules modelled using different formalisms. In Liu et al. (2022), five complementary combinations of modelling formalisms are used for biological systems: *hybrid discrete‐continuous* methods, often used for intracellular modelling as above; *hybrid stochastic‐deterministic* methods, introducing random delays; *hybrid flux balance analysis* methods, integrating metabolic, regulatory and signalling networks; *hybrid logic‐quantitative* methods, combining quantitative methods with Boolean networks or fuzzy logic; and *spatial hybrid* methods, combining spatial stochastic and deterministic models to bridge three scales: time, abundance and space (Widmer & Stelling, 2018).

More formally, *hybrid dynamical systems* often combine continuous *state* and *control* variables plus a discrete *mode* variable, the transition of which is determined by *guard* conditions defined on the continuous variables (Henzinger, 1996). In *switched systems*, the mode activates, deactivates and combines modules of a hierarchical model (Branicky, 1994; Shorten et al., 2007). In this framework, phenomenological models and GEMs can be coupled in a flexible and rigorous way. A good example can be found in Assar et al. (2014), that switches between two ODE models (Coleman et al., 2007; Scaglia et al., 2009) and a dFBA model of wine fermentation kinetics (Pizarro et al., 2007) according to the system state.

## FROM ENVIRONMENTAL SAMPLES TO FUNCTIONAL AND TAXONOMIC DIVERSITY

The preceding has taken a high‐level view of the modelling process and the insights models can provide, and has assumed that the collection of models for a community accurately reflects the taxonomic composition of its members and the biomolecular functions they provide. These will vary greatly between environments and from sample to sample: different species provide different functions, and within those species strain differences and horizontal gene transfer will further affect gene content. In this section, we consider how molecular identification of taxonomic and functional diversity is employed to build accurate characterisations of real communities.

Two DNA sequencing approaches are used when building metabolic models from environmental samples (Pérez‐Cobas et al., 2020). *Amplicon sequencing* of marker genes characterises taxonomic diversity within a sample and can be used to infer functional profiles, map community members to existing models (Heinken & Thiele, 2022) and select genomes prior to GENRE reconstruction. *Whole metagenome shotgun sequencing* characterises both taxonomic and functional diversity within a sample by determining the gene content of community members, and can be used to infer new GENREs of novel species and strains. In both approaches, *third‐generation sequencing* (TGS) using long reads can complement, or advantageously replace, NGS using short reads.

Amplicon sequencing and shotgun metagenomic sequencing have demonstrated the high diversity of microbial communities, for bacteria and archaea, but also eukaryotes such as fungi (Tedersoo et al., 2014).

### Amplicon sequencing

A good *marker gene* is universally present in target populations and taxonomically robust because its infrequent mutation is not biased by functional selection. Typical choices for marker genes are 16S ribosomal RNA genes in prokaryotes, and either 18S ribosomal RNA genes or different lineage‐specific markers in eukaryotes (Bradley et al., 2016; Burki et al., 2020; Earl et al., 2018; Johnson et al., 2019; Tekle et al., 2010). For a given environmental sample, the variable regions of the selected marker genes are amplified using PCR and later sequenced. Until recently, inexpensive short‐read NGS sequencing has been used exclusively (Bukin et al., 2019; Kim et al., 2021), although this limits the size of the variable region that can be used, may be subject to amplification bias and may limit taxonomic resolution to phylum and genus due to 16S polymorphism (Knight et al., 2018; Mitchell et al., 2020; Sieber et al., 2018). Long‐read TGS sequencing of full‐length 16S rRNA has recently been shown to reduce bias, broaden the choice of marker genes and increase taxonomic resolution to species and subspecies (de Muinck et al., 2017; de Oliveira Martins et al., 2019; Johnson et al., 2019).

Amplicon sequences are clustered into *operational taxonomic units* (OTUs) or into *amplicon sequencing variants* (ASVs) that provide ‘species‐like’ delineation of organisms and estimations of relative abundance. Bioinformatic pipelines for amplicon sequencing analysis are reviewed in Hakimzadeh et al. (2023). While the functions present in a microbiome cannot be directly identified by amplicon sequencing (Almeida et al., 2021), approximations may be indirectly inferred using phylogenetic placement of markers (Bowman & Ducklow, 2015; Douglas et al., 2020), alignment (Narayan et al., 2020; Wemheuer et al., 2020) or incorporation of biological knowledge in the form of taxonomic and functional profiles (Jing et al., 2021; Mallick et al., 2019). Nonetheless, limitations resulting from the importance of the accessory genome in a species with respect to its core genome will impact the accuracy of the predictions.

Building mechanistic models of metabolism requires accurate taxonomic classification, both for identifying existing genomes and GENREs and characterising diversity. The KSGP database (Grant et al., 2023) combines the GTDB unified taxonomy (Parks et al., 2020) with a collection of environmentally sampled full‐length sequences, to substantially increase taxonomic assignment of environmental samples and links to reference genomes. Resolution within reference genomes is typically limited to species and will miss most strain‐specific variability.

### Whole metagenomic sequencing

Whole metagenomic sequencing uses total DNA extracted from the environmental sample to build a complete picture of the genomes of the organisms present in the sample. Also called *untargeted*, *shotgun*, *random* or *WGS* sequencing, whole metagenomic sequencing does not target a specific amplified marker gene. While sequencing a larger amount of genetic material comes with increased expense, it avoids many of the shortcomings of amplicon sequencing: it can taxonomically resolve species, and even strains if sufficient data; it can identify the genes carried by species on a sample‐specific basis, even for unknown taxa; and it can infer microbial functions including strain‐specific variants in the accessory genome (Djemiel et al., 2022; Hildebrand, 2021; Hildebrand et al., 2019, 2021).

Table 3 summarises the short‐read and long‐read sequencing technologies used for assembling genomes, and reconstructing metagenomes and MAGs (MetaHIT Consortium et al., 2015; Nielsen et al., 2014; Pan et al., 2023). As above, long TGS reads can significantly improve results (Kim et al., 2022). PacBio HiFi long‐read sequencing has low error rates and provides near‐finished microbial genomes from pure cultures or metagenomes, albeit at relatively high cost (Amarasinghe et al., 2020). Oxford Nanopore (ONT) sequencing produces even longer reads at higher throughput on average, but with higher rates of homopolymer indels that may lead to gene calling errors (Watson & Warr, 2019). Combining long‐read ONT sequencing with short reads for post‐assembly error correction is a common compromise (Delahaye & Nicolas, 2021), although more recently, HiFi PacBio metagenomic assembly using metaMDBG (Benoit et al., 2023) has improved on that. Both PacBio and ONT are rapidly advancing their technological development, and due to improvements in sequence length, quality and throughput will likely replace short‐read NGS technologies entirely in the near term.

**TABLE 3:** Main short‐read and long‐read metagenomic sequencing technologies.

Method	Sequencing platform	Read lengths (bp)	Throughput	Specifications	Applications advantages/pitfalls
Short‐read metagenomics	Illumina Miseq system	2 × 300	Low	1–25 M reads, up to 15 Gbp >70% bases higher than Q30	Taxonomic profiling, Relatively low data processing requirements
	Illumina Novaseq 6000	2 × 150	High	1.6B–40B reads, up to 3 Tb, ≥85% of bases higher than Q30	Taxonomic and functional profiling, Genome assembly and binning; High throughput, low cost for production‐scale genomics
	Pacific Biosciences Onso	2 × 150	Moderate	120–150 Gbp ≥90% bases Q40	Taxonomic and functional profiling Genome assembly and binning Identification of variants 15× higher accuracy than other short read platforms High accuracy in characterisation of homopolymers Higher costs/bp
Long‐read metagenomics	Pacific Biosciences Sequel II and IIE	Typically 10–30 k	Moderate	3M reads typically Q20–Q40 reads	Taxonomic and functional profiling, Genome assembly and binning Can reconstruct circular MAGs High sample input: ~2 μg Full‐length 16S rRNA seq
	Pacific Biosciences Revio system	~10 to ~30k	Moderate	Up to 90 Gbp Q30‐Q40	Higher HiFi read accuracy with DeepConsensus base calling, methylation calling High sample input: ~2 μg ‐ Full‐length 16S rRNA seq
	Oxford Nanopore Technologies Promethion R10 series	On average ~10k to ~60k, strongly dependent on DNA extraction	Moderate	~50 Gb >median Q20 most reads, ~Q30 (99.9%) for Duplex reads	Very long reads Transportable technology Low sample input: ~1 μg Less polishing needed than R9 series Read quality still lower than PacBio

Abbreviations: bp, base pair; ONT, Oxford Nanopore Technologies; PacBio, Pacific Biosciences; Q, quality score logarithmically related to the base calling error probability, Q20: 1%, Q30: 1‰ etc. Read length is subject to variation.

### Metagenomic strains

In order to fully understand microbial communities, it is necessary to resolve them into individual strains, the fundamental unit of microbiological diversity (Hildebrand, 2021; Segata, 2018). *Escherichia coli*, which has a highly variable genome, is a well‐known example of the importance of strains in functional characterisation: some are harmless commensals while others are harmful pathogens (Leimbach et al., 2013). Resolving metagenomic data at the strain level brings a new dimension to functional prediction and to the comparison of the metabolic potential of samples. A *metagenomic strain* is a set of clonal descendants of a single cell, with such low levels of recombination with other strains that it can be distinguished genetically from them. Members of a metagenome strain may differ substantially, particularly in the rapidly evolving accessory genome (Maistrenko et al., 2020). Reconstructing metagenomic strains is difficult since sequencing depth may be insufficient for genome reconstruction and conspecific strains with overlapping genetic regions may be present. Strain‐resolved genomes can be determined by sequencing cultured isolates or single cells, but the former are not representative of the community and the latter remain too expensive and low‐throughput for many applications. For these reasons, there is a practical need for efficient methods that can profile microbial communities at high genomic resolution (Bickhart et al., 2022).

*Reference‐based methods* for delineating microbial strains in metagenomes rely in general on aligning metagenomic reads to a reference database and inferring strain identity based on single nucleotide polymorphisms (SNPs) (Albanese & Donati, 2017; Fischer et al., 2017; Scholz et al., 2016; Silva et al., 2021; Truong et al., 2017). These methods identify gene family presence, strain identification for dominant strains and abundance estimates.

*Strain‐resolved metagenomics* compares de novo reconstructed MAGs from different metagenomes to each other, without use of a reference database. Gene catalogues can be used to trace core genes in MAGs across samples, which can be compared to create within‐species strain‐resolved phylogenies of metagenomes and avoid pan genome‐induced phylogenetic idiosyncrasies (Hildebrand et al., 2021).

When investigating conspecific strains in the same metagenomic sample, specialised tools model SNP frequencies across assemblies to infer bacterial haplotypes and identify conspecific strains, even to providing nearly complete pan genome reconstructions, or delineate strains using TGS data of low‐complexity metagenomes (Quince, Delmont, et al., 2017; Quince et al., 2021; Vicedomini et al., 2021). Metagenomic strains can be mapped to GENREs, as shown in Basile, Heinken, et al. (2023) where longitudinal community models were built to describe metabolic changes over time of a well‐sampled microbiome.

Single‐cell technologies can also rise to the challenge of recovering genomes of low abundant strains and microbial dark matter (see Lloréns‐Rico et al., 2022 for review), especially through the generation of *single amplified genomes* (SAGs) and the promise of modelling rare species (Arikawa et al., 2021; Bowers et al., 2022; Lyalina et al., 2022; Zheng et al., 2022). Single‐cell transcriptomics also promises to contribute to context‐specific models (Hrovatin et al., 2021).

## FROM METAGENOMIC DATA TO ASSEMBLED GENOMES

### First steps of data processing and assembly‐free analysis

**FIGURE 4:**
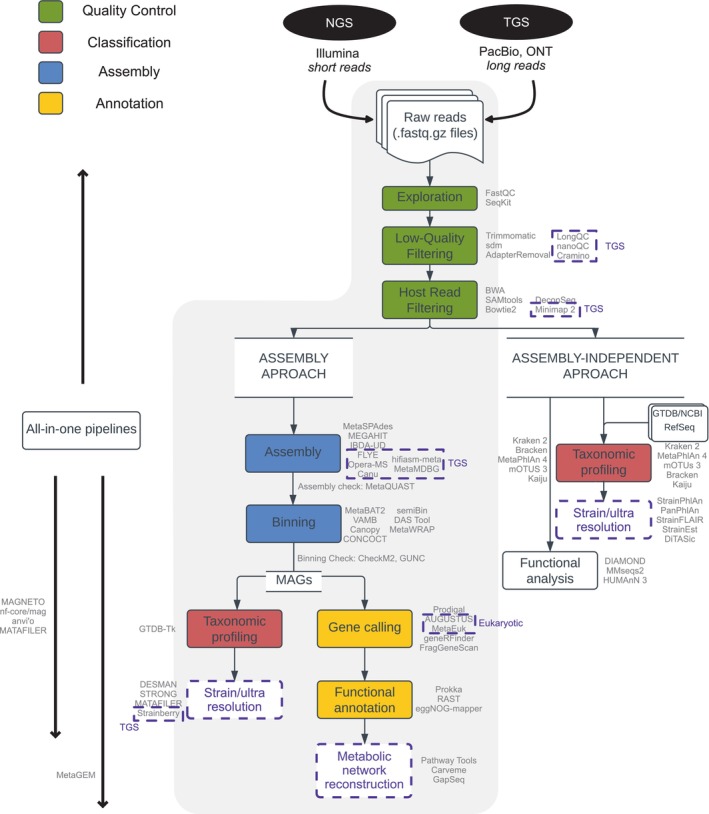
Metagenomic sequencing and data processing. After quality control, shotgun metagenomic reads can enter an assembly pipeline or an assembly‐independent profiling pipeline. The assembly pipeline is highlighted as it permits the reference‐free reconstruction of MAGs and subsequently GENRE reconstruction that directly survey the functions present in the ecosystem. Examples of tools associated with each step are indicated next to the box. Tools or steps related specifically to TGS, eukaryotic metagenomics and strain resolution are highlighted with dotted boxes.

A standard metagenomic analysis workflow comprises: (1) sample collection, DNA extraction and DNA sequencing; (2) data pre‐processing and read curation, including quality control and filtering; (3) taxonomic profiling via either ‘read‐based’ or ‘assembly‐based’ approaches; and (4) further downstream analyses of genomic and functional characteristics, such as gene prediction, and functional or taxonomic annotation (Quince, Walker, et al., 2017) (Figure 4). In this simplified workflow, (2), (3) and (4) are typically executed in a single pipeline, or can be adjusted to the analytical needs of a project. A crucial decision in the bioinformatic analysis is the choice between reliance on assemblies, or on analysing metagenomes independent of this. Assemblies will in most cases require further computational resources and user expertise, but offer in exchange a much richer datascape to explore in the follow‐up analysis. For example, to build models of metabolism and GENREs in particular, sequence assembly and MAG reconstruction are required to delineate the functional role of bacterial species in the community.

Once sequences for the chosen samples have been acquired, they are screened; trimmed to remove low‐quality ends, bar codes, adaptors and technical artefacts; and filtered for quality (Table 4). Specific tools are available for quality filtering of long‐read sequencing data (Coster et al., 2018; Coster & Rademakers, 2023; Fukasawa et al., 2020; Li, 2018). Host‐associated samples such as gut and oral metagenomes may be contaminated with reads originating in the host organism that should be removed to avoid chimeric assemblies or false‐positive assignment to bacterial taxa (Li et al., 2009; Li & Durbin, 2009; Schmieder & Edwards, 2011).

**TABLE 4:** Main sequence analysis methods.

OTU clustering	
QIIME 2	Estaki et al. (2020)
DADA2	Callahan et al. (2016)
LotuS2	Özkurt et al. (2022)
Function inference	
PICRUSt2	Douglas et al. (2020)
Paprica	Bowman and Ducklow (2015)
Tax4Fun2	Wemheuer et al. (2020)
Piphillin	Narayan et al. (2020)
Metabolic features	
MelonnPan	Mallick et al. (2019)
Meta‐Apo	Jing et al. (2021)
Taxonomic classification	
KSGP	Grant et al. (2023)
GTDB	Parks et al. (2020)
Adapter removal	
Trimmomatic	Bolger et al. (2014)
sdm	Hildebrand et al. (2014)
AdapterRemoval	Schubert et al. (2016)
Quality control	
FastQC	Andrews et al. (2010)
SeqKit	Shen et al. (2016)
Long read analysis	
LongQC	Fukasawa et al. (2020)
nanoQC	Coster et al. (2018)
Cramino	Coster and Rademakers (2023)
Minimap2	Li (2018)
Taxonomic filtering	
MetaPhlAn4	Blanco‐Miguez et al. (2022)
mOTUs3	Ruscheweyh et al. (2022)
Functional profiling	
Kaiju	Menzel et al. (2016)
DIAMOND	Buchfink et al. (2014)
MMseqs2	Steinegger and Söding (2017)
Short read assembly	
metaSPAdes	Nurk et al. (2017)
IDBA‐UD	Peng et al. (2012)
MEGAHIT	Li et al. (2015)
Long read assembly	
MetaFlye	Kolmogorov et al. (2020)
Canu	Koren et al. (2017)
Opera‐MS	Bertrand et al. (2019)
hifiasm‐meta	Feng et al. (2022)
MetaMDBG	Benoit et al. (2023)
Assembly evaluation	
MetaQUAST	Mikheenko et al. (2016)
Gene prediction	
Prodigal	Hyatt et al. (2010)
MetaEuk	Karin et al. (2020)
Reference gene catalogues	
CD‐HIT	Fu et al. (2012)
MMseqs2	Steinegger and Söding (2017)
Binning	
CONCOCT	Alneberg et al. (2014)
MaxBin 2	Wu et al. (2016)
MetaBAT 2	Kang et al. (2019)
Bin consolidation	
VAMB	Nissen et al. (2021)
SemiBin2	Pan et al. (2022)
MetaWRAP	Uritskiy et al. (2018)
DAS Tool	Sieber et al. (2018)
Chimerism and contamination	
CheckM	Parks et al. (2015)
GUNC	Orakov et al. (2021)
MAG creation pipelines	
MAGNETO	Churcheward et al. (2022)
nf‐core/mag	Krakau et al. (2022)
anvi'o	Eren et al. (2021)
MATAFILER	Hildebrand et al. (2019)
MetaGEM	Zorrilla et al. (2021)

While assembly‐based metagenomics is the approach of choice for building mechanistic models of metabolism, we briefly present here assembly‐independent alternatives for taxonomic functional inference. Preprocessed metagenomic reads can also be used to infer the microbial taxonomic and functional potential composition in the sequenced samples, using DNA, marker gene or protein‐based approaches. Read‐based taxonomic profilers estimate the relative abundance of microbes in an environmental sample by aligning the preprocessed sequencing reads to a reference sequence database such as GTDB (Parks et al., 2020) or NCBI RefSeq (O'Leary et al., 2016), using either mapping (Langmead & Salzberg, 2012) or k‐mer matching (Lu et al., 2022). The reference genome search space can be drastically reduced by including only conserved marker genes that are specific to certain taxonomic groups (Blanco‐Miguez et al., 2022; Ruscheweyh et al., 2022). Read‐based profilers can also be used for functional inference (Beghini et al., 2021). Protein‐based functional profilers use six‐frame translations into amino acid sequences (Buchfink et al., 2014; Menzel et al., 2016; Steinegger & Söding, 2017), with the advantage of greater sensitivity because of the lower mutation rates in amino acids, but at the cost of significant increase in run time (Ye et al., 2019).

The choice of reference database plays an important role in read‐based taxonomic profiling. Microbes lacking a reference genome will remain undetected, which is a problem in metagenomics as much of the microbial diversity is not yet represented in reference databases (Solden et al., 2016). In addition, the read‐based approach does not permit the direct association of microbial diversity to metabolic functions. While it can be used for building a community‐wide model as a super‐organism associated with a metagenome, a common protocol is instead to assemble the reads and reconstruct genomes.

### Metagenomic assemblies

Reconstructing microbial genes or partial genomes by assembling sequences from environmental samples overcomes limitations of reference‐based metagenomic approaches, but requires significant sequencing read depth and bioinformatics expertise. While shallow shotgun sequencing (ca. 0.5 million short reads per sample) can be enough to capture the taxonomic and functional diversity of microbiome samples at relatively low cost, it does not provide sufficient coverage for de novo assembly of novel genes and genomes (Lapidus & Korobeynikov, 2021).

Quality filtered reads can be directly assembled using metagenomic‐specific assemblers, designed for short reads, long TGS reads, or in hybrid combinations (Table 4), see Brown et al. (2021) for review. Assemblers that specifically target high‐fidelity long reads perform well in general on middling‐complex communities such as the gut microbiome and have an acceptable computational efficiency for large‐scale metagenomes (Benoit et al., 2023; Feng et al., 2022). When sampled metagenomes are expected to be highly similar to each other, as in, for example, time‐series data of the same ecosystem with reasonably stable strain persistence, co‐assemblies of time‐series samples can be performed to increase assembly quality (Hildebrand et al., 2019, 2021).

The drawback of assembly‐based metagenomics is the high potential for errors in the assembly, and the inability to include a large part of the reads in the assembly, especially those from low‐abundant genomes in highly diverse, complex ecosystems. The fraction of non‐assembled reads depends on several factors, especially sequencing depth and microbiome diversity, and can range from <5% in gut to >90% in soil metagenomes (Bahram et al., 2018; Hildebrand et al., 2019). Even with deeper short‐read shotgun sequencing, de novo assembly remains a challenge due to the presence of repetitive DNA regions, shared genomic regions between strains and the complexity of microbial communities with unknown and uneven representation of both diverse and closely related strains (Ayling et al., 2020; Somerville et al., 2019). TGS can overcome many of the difficulties in metagenomic assembly by spanning repetitive sequences and highly conserved regions. Before the development of the latest low‐error technologies, the need for short‐read data to overcome high error rates and low coverage made this approach costly and complex (Kim et al., 2022). The low error rate of PacBio HiFi reads constitutes a valuable alternative with high quality, circular genomes frequently being reported in the data currently available (e.g. Kim et al., 2022, Table 3).

#### Gene catalogue creation and gene prediction

Genes can be predicted on contigs, but having the complete sequence of a gene and its context makes its functional and taxonomic assignment and its annotation much easier and more reliable, facilitating the construction of community‐wide GENREs (Tamames & Puente‐Sánchez, 2018).

To capture the set of proteins represented in a set of metagenomes, one can generate reference gene catalogues. These typically start with the genes predicted on contigs from metagenomic assemblies that are subsequently clustered, for example, at 95% identity, in order to remove trivial differences between sequences due to fragmentary data (e.g. genes that miss the start or stop codons), sequencing errors or small, strain‐level variations (Qin et al., 2010). The clustering can be performed either at the nucleotide or amino acid level (Fu et al., 2012; Steinegger & Söding, 2017); the former provides greater resolution for taxonomic classification, whereas the latter is better suited for identifying functionally relevant similarities between distant orthologues (Commichaux et al., 2021; Wang & Jia, 2016).

The underlying goal of the clustering process is to reproducibly group together sequences that have the same function or the same taxonomic origin, thereby identifying the genes from which the sequences are derived in a way that is consistent across samples. Each cluster is typically represented by one sequence, either a representative selected from the sequences clustered together or a sequence that represents the consensus of the clustered sequences. Cluster representatives can be used in a broad range of sequence‐based analyses (e.g. database searches, function or structure predictions), and also to estimate the relative abundance of a single gene across metagenomes using a gene catalogue (Commichaux et al., 2021; Plaza Oñate et al., 2019). As gene catalogues reduce the redundancy of the data, they can be used as a basis of exhaustive surveys that compare the metabolic potential of metagenomic samples by associating metabolic functions with the genes.

#### Creation of metagenome‐assembled genomes (MAGs)

Most contigs obtained from assemblies are only fragments of pro‐ or eukaryotic genomes. TGS permits the assembly of near‐complete genomes for abundant species. For less abundant organisms and when using short‐read sequencing data, it is necessary to add a *binning step* that sorts contigs into ‘bins’ according to the genome they likely originate from, based on assumptions of shared sequence composition, orthologous linkage data and shared co‐abundance within and between samples (Alneberg et al., 2014; Kang et al., 2019; Nissen et al., 2021; Pan et al., 2022; Wu et al., 2016). VAMB (Nissen et al., 2021) in particular allows splitting clusters into sample‐specific bins according to their sample of origin, an approach termed ‘multisplit’, which is useful for comparing pan genomes of conspecific strains. *Bin consolidation* assimilates bins created by different tools into a set of refined bins (Sieber et al., 2018; Uritskiy et al., 2018).

Genome bins of sufficient quality to approximate a genome are commonly called ‘MAGs,’ for *metagenome‐assembled genome*. Popular measures of MAG quality are the genome *completeness*—a desirable measure of the proportion of genome recovered for the species of interest—and genome *contamination*, the undesirable amount of genome fragments that are of different species origin and been mistakenly binned together (Chen et al., 2020). Tools exist for quality assessment using single‐copy marker genes as well as detection of contamination and chimeric assemblies (Table 4). A MAG is said to be of high quality when it has over 90% of the expected count of SCGs with less than 5% redundancy of their prevalence, and containing assemblies of ribosomal RNA (rRNA) and transfer RNA (tRNA) genes (Bowers et al., 2017). Abundance of MAGs in a sample can be estimated using (1) mapping raw metagenomic reads on the newly created MAG and (2) extracting informative marker genes for each MAG to which then either reads are mapped, or their abundance is derived from a gene catalogue (as used in Hildebrand et al. (2021)).

Even though building MAGs is data‐ and computation‐intensive, they provide significant insights into the genomic and functional diversity found in different habitats (Almeida et al., 2019; Kashaf et al., 2022). MAGs are contig‐based clusters and thus preserve the relative order of genes along contigs, which is of importance as genes found in close physical proximity may encode biosynthetic pathways for the synthesis of specialised metabolites (Almeida et al., 2021). Dozens of high‐quality MAGs can be reconstructed from short‐read data of a single gut metagenome, but rarely as continuous, circular genomes. Here, TGS technologies can potentially yield dozens or hundreds of closed genomes without binning, markedly increasing the quality of culture‐independent genome reconstructions (Kim et al., 2022).

All‐in‐one pipelines combine tools to reconstruct MAGs directly from sequencing reads (Churcheward et al., 2022; Eren et al., 2021; Hildebrand et al., 2019; Krakau et al., 2022). MAGs can be used for GENRE reconstruction and thus enable metabolic modelling, as in Bernardini et al. (2022) where the authors performed FBA simulations to examine interspecies metabolic interactions in anaerobic digestion. MetaGEM (Zorrilla et al., 2021) is a MAG reconstruction pipeline that directly bridges the gap between metagenomics and metabolic modelling, as it proposes to add additional pipeline steps for the automatic reconstruction of GENREs using Carveme and metabolic interaction inference using SMETANA. While all‐in‐one pipelines are valuable, the difficulty of metagenome assembly combined with the complexity of microbial ecosystems maintains the need for curation and additional analyses, especially to capture certain taxonomic clades such as eukaryotes (see subsection “Extending Metagenomic Assembly to Eukaryotes”).

#### Extending metagenomic assembly to eukaryotes

Along with prokaryotes, eukaryotes are significant drivers of microbiomes. For instance, autotrophic and mixotrophic protists fix carbon in aquatic environments, and heterotrophic protists catalyse nutrient cycling in aquatic and terrestrial ecosystems (Massana & López‐Escardó, 2022; Worden et al., 2015). Fungi are much more prevalent in soil and aquatic ecosystems (Bahram et al., 2020) and play essential roles in global nutrient cycles and the plant rhizosphere (Tedersoo et al., 2014). Much can be learned about the diversity and role of eukaryotes in our environment from eukaryotic MAG retrieval, but several technical hurdles prevent this currently: (1) eukaryotic genomic complexity, due to larger genomes, multiple chromosomes and the many non‐coding and repetitive regions, complicates both metagenome assembly and MAG retrieval; (2) commonly studied environments, such as the human gut, contain only very few microeukaryotes, at typically low abundance (Bahram et al., 2020; Tito et al., 2019); and (3) there is a bias in the currently available metagenomic computational tools towards the study of bacterial and archaeal members of the community (Massana & López‐Escardó, 2022; Pronk & Medema, 2022).

The difference in gene structure between prokaryotes and eukaryotes is a major obstacle to the identification and annotation of eukaryotic genes. Prokaryotic tools applied to eukaryotic contigs tend to predict incomplete, erroneous and discontinuous genes and thus protein sequences. Consequently, eukaryotic proteins are neglected in microbiome data, with a risk of being truncated and assigned unreliable annotations (Karlicki et al., 2022; Pronk & Medema, 2022). Most public metagenomic repositories use annotation pipelines tailored for prokaryotes, regardless of the taxonomic origin of contigs (Lind & Pollard, 2021).

Recent methodological developments tackle this challenge, through the identification of eukaryotic contigs and genes (Karlicki et al., 2022; Pronk & Medema, 2022; Stanke et al., 2004) and their subsequent annotation, for instance with MetaEuk (Karin et al., 2020). A few examples of eukaryotic MAGs have been reported, but these relied either on metagenomes with a higher fraction of eukaryotes present (such as e.g. Duncan et al., 2022), or on human gut samples that were clearly dysbiotic and had an overgrowth of a single fungal species (Olm et al., 2019). These examples demonstrate some of the difficulties associated with investigating eukaryotic metagenomes and that methodological challenges remain. Nonetheless, the recovery of eukaryotic genomes in microbiomes has a major ecological interest and it will be facilitated by TGS.

## IMPORTANCE OF DATA REUSE AND REPURPOSING IN METAGENOMICS AND METABOLIC MODELLING

The data pipeline from the reads to the model encompasses a long list of steps, tools and parameters that scientists may use and combine in the process. This raises important questions related to the reproducibility of the analysis outcomes, and the traceability of the data and processes involved. We provide below an overview of the importance of these aspects in the context of the topics treated in the review.

Characterising microbial communities requires data, data are acquired in context, and context provides key clues for deciphering the function of a gene or an organism. *Metadata* are descriptions of, and facts about, data and other research outputs. Metadata for genomic and metagenomic data from environmental samples can describe the context of the source material providing the data, including space, time, habitat, characteristics of the environment, biotic relationships; as well as sequencing and assembly methods (Yilmaz et al., 2011).^1^ For metabolic modelling, metadata can include references to genomes, traces of curation and tools used for reconstruction (Aite et al., 2018). Indeed, the computational processes that produce research outputs are also performed in a context, which provides key information for reproducing, evaluating and reusing those research outputs. Metadata can therefore also describe computational processes across an analytic stack consisting of input data, tools, reports, pipelines and publications (Leipzig et al., 2021).

Data and reporting standards must be used in order to guarantee interoperability, reuse and repurposing. Reuse of publicly available raw sequences of metagenomes or metagenomic assemblies is very common thanks to dedicated resources such as MGnify (Richardson et al., 2022), and MAGs as well are made available in databases. The Genomic Standards Consortium (GSC) has formalised minimum reporting standards for genomes, metagenomes—minimum information about metagenomic sequence (MIMS)—and marker gene sequences with these purposes in mind (ten Hoopen et al., 2017; Yilmaz et al., 2011). In particular, the minimum information about a metagenome‐assembled genome (MIMAG) (Bowers et al., 2017) is a community‐developed standard for reporting bacterial and archaeal genome MAGs.

Metabolic modelling is not exempt from considerations related to traceability. Given the variety of tools suitable for GENRE generation, it is important to promote their sharing and standardisation. The use of standardised nomenclatures for GENREs reconstructed in different labs, such as in the BiGG database (Norsigian et al., 2020), is crucial for GENRE comparison and construction of multispecies GEMs. Indeed, a recurring problem lies in the reconciliation of identifiers across databases, for which MetaNetX provides identifier mapping resources (Moretti et al., 2016). A standard format for the representation and exchange of GENREs but also cell signalling pathways and other systems biology models is the Systems Biology Markup Language (Hucka et al., 2018). This declarative format based on XML (eXtensible Markup Language) not only permits the representation of the metabolic network but also includes mathematical parameters used for its simulation. However, the use of this format does not guarantee by itself the quality of the model contents, its reusability with other tools nor the compatibility of the identifiers with different databases. MEMOTE is a community‐based test suite for the assessment of GENRE quality, representing a valuable effort in the direction of standardisation, reproducibility and interoperability (Lieven et al., 2020).

Widespread adoption of *FAIR* guidelines (Wilkinson et al., 2016) is a further driver of the formalisation of data and metadata, since the latter are essential for finding and reusing the former. Going beyond this basic requirement, a key opportunity is to promote *reuse* and *repurposing* of research objects (Bechhofer et al., 2013). Capturing the computational processes that produce research objects, is critical for reproducibility (Cohen‐Boulakia et al., 2017; Leipzig et al., 2021); identify the needs—and challenges—of improved reproducibility; and define criteria for ‘reproducibility‐friendly’ scientific workflow systems.

## CONCLUSION AND FUTURE PROSPECTS

Improvements in genome reconstruction from metagenomic data have made it possible to build data‐informed metabolic models for microbial communities. These mechanistic models describe the phenomenological behaviour of the community, but furthermore capture the metabolic functions of under‐represented species and strain‐specific metabolic variations that play an important role in the community disproportionate to their abundance. Third‐generation sequencing has proved to be a key technology for answering challenges in metagenomics; providing better recovery of eukaryotic genomes; allowing strain‐level analysis and pangenomics; and meeting algorithmic challenges in genome delineation. Improved metagenomics leads to improved metabolic models that more fully describe communal functions as well as the added value of individual species and strains. The translation of the latest technologies into routine experimental design will likely take years, however; algorithmic developments addressing these same questions for NGS short‐read metagenomics are still needed in the meantime.

Metagenomics is the –omics data that contributes the most to community‐wide metabolic models, in the same way that genomics contributes to models of individual species. Integrating transcriptomics and metabolomics to build *integrative models*, following a systems biology and systems ecology approach, is necessary to fully comprehend the (eco)system. Even if these data are not obtained systematically, they can be valuable for calibrating metabolic models.

Depending on the complexity of the ecosystem and despite the use of TGS, the number of MAGs retrieved from environmental samples is today subpar, and the proportion of raw reads they make use of can remain limited (Pessi et al., 2022). Those limitations may prevent MAG‐derived metabolic models from accurately reflecting the complete functional diversity of the microbiome. Continuing efforts in developing efficient algorithms for MAG reconstruction must be synergetic with sequencing technique improvements.

Throughout this review, we contrasted phenomenological and mechanistic models as complementary ways of describing the complex behaviour of microbial communities. As alluded to in Section “Towards Genomics‐Informed Phenomenological Models of Microbial Communities”, hybrid models are a promising avenue for reconciling both approaches. They could combine coarse‐grained but highly scalable phenomenological modelling with limited fine‐grained modelling of selected systems, such as the often‐overlooked secondary metabolism. Developing a hybrid paradigm would combine the advantages of the two approaches and greatly contribute to building complete understanding of natural microbial communities.

## AUTHOR CONTRIBUTIONS

**Klara Cerk:** Visualization (equal); writing – original draft (equal); writing – review and editing (supporting). **Pablo Ugalde‐Salas:** Visualization (equal); writing – original draft (equal); writing – review and editing (supporting). **Chabname Ghassemi Nedjad:** Writing – original draft (equal); writing – review and editing (supporting). **Maxime Lecomte:** Visualization (supporting); writing – original draft (equal); writing – review and editing (supporting). **Coralie Muller:** Writing – original draft (equal); writing – review and editing (supporting). **David J. Sherman:** Writing – original draft (equal); writing – review and editing (lead). **Falk Hildebrand:** Supervision (equal); visualization (equal); writing – original draft (equal); writing – review and editing (lead). **Simon Labarthe:** Conceptualization (equal); supervision (equal); visualization (equal); writing – original draft (equal); writing – review and editing (lead). **Clémence Frioux:** Conceptualization (equal); supervision (lead); visualization (equal); writing – original draft (equal); writing – review and editing (lead).

## CONFLICT OF INTEREST STATEMENT

The authors declare no conflict of interest.

## Footnotes

^1^

https://schema.datacite.org.
